# Comparison of tolerance of four bacterial nanocellulose-producing strains to lignocellulose-derived inhibitors

**DOI:** 10.1186/s12934-017-0846-y

**Published:** 2017-12-21

**Authors:** Xiaozhou Zou, Guochao Wu, Stefan Stagge, Lin Chen, Leif J. Jönsson, Feng F. Hong

**Affiliations:** 1 State Key Laboratory for Modification of Chemical Fibers and Polymer Materials, Donghua University, Shanghai, 201620 China; 2 China-Sweden Associated Research Laboratory in Industrial Biotechnology, College of Chemistry, Chemical Engineering and Biotechnology, Donghua University, Shanghai, 201620 China; 3 Department of Chemistry, KBC Chemical-Biological Centre, Umeå University, 901 87 Umeå, Sweden; 4 Group of Microbiological Engineering and Industrial Biotechnology, College of Chemistry, Chemical Engineering and Biotechnology, Donghua University, Shanghai, 201620 China

**Keywords:** Bacterial cellulose, Lignocellulose, Inhibitors, Acetic acid bacteria, *Komagataeibacter xylinus*

## Abstract

**Background:**

Through pretreatment and enzymatic saccharification lignocellulosic biomass has great potential as a low-cost feedstock for production of bacterial nanocellulose (BNC), a high value-added microbial product, but inhibitors formed during pretreatment remain challenging. In this study, the tolerance to lignocellulose-derived inhibitors of three new BNC-producing strains were compared to that of *Komagataeibacter xylinus* ATCC 23770. Inhibitors studied included furan aldehydes (furfural and 5-hydroxymethylfurfural) and phenolic compounds (coniferyl aldehyde and vanillin). The performance of the four strains in the presence and absence of the inhibitors was assessed using static cultures, and their capability to convert inhibitors by oxidation and reduction was analyzed.

**Results:**

Although two of the new strains were more sensitive than ATCC 23770 to furan aldehydes, one of the new strains showed superior resistance to both furan aldehydes and phenols, and also displayed high volumetric BNC yield (up to 14.78 ± 0.43 g/L) and high BNC yield on consumed sugar (0.59 ± 0.02 g/g). The inhibitors were oxidized and/or reduced by the strains to be less toxic. The four strains exhibited strong similarities with regard to predominant bioconversion products from the inhibitors, but displayed different capacity to convert the inhibitors, which may be related to the differences in inhibitor tolerance.

**Conclusions:**

This investigation provides information on different performance of four BNC-producing strains in the presence of lignocellulose-derived inhibitors. The results will be of benefit to the selection of more suitable strains for utilization of lignocellulosics in the process of BNC-production.

## Background

Bacterial nanocellulose (BNC) is a nano-fibrillated pure cellulose synthesized by bacteria. It is a high value-added biomaterial with a number of characteristic properties, and has great potential for many applications. BNC-producing bacteria include several genera, such as *Acetobacter*, *Achromobacter*, *Aerobacter*, *Agrobacterium*, *Alcaligenes*, *Pseudomonas*, *Rhizobium*, *Sarcina,* and *Zoogloea*. *Komagataeibacter xylinus* (formerly *Gluconacetobacter xylinus* or *Acetobacter xylinum*) is an example of a species that efficiently produces BNC [1]. BNC has unique physicochemical features, such as high purity, high degree of crystallinity, high degree of polymerization, large surface area, high tensile strength in wet state, high absorbency, good biocompatibility, non-toxicity, mechanical stability, and high moisture content [2]. Therefore, BNC has a wide application area that includes textile industry, mining, waste treatment, paper industry, and food production [3], but especially biomedical materials [4] including vascular grafts [5, 6], tissue-engineering scaffolds [7], carrier for drugs and delivery of other bioactive compounds [8], and wound dressing [9, 10].

To decrease the production cost and increase the quality of BNC, residual renewables from industry and agriculture, for instance lignocellulosic residues from industrial crops, have been studied as potential cost-effective feedstocks for production of BNC. Such potential feedstocks include konjak glucomannan [11], sugarcane molasses [12, 13], wheat straw [14, 15], rice bark [16], corncob [17], waste textiles [18–20], waste fiber sludge [21], spruce wood residue [22], and cashew tree residues [23]. To be useful for preparation of culture media for BNC-producing bacteria, most lignocellulosic residues need to be pretreated for efficient saccharification of cellulose to fermentable sugars. Due to pretreatment at high temperatures and low pH, many lignocellulose-derived compounds appear in the hydrolysates [24, 25]. These compounds include aliphatic carboxylic acids, furan aldehydes, and phenolic compounds and other aromatics [26]. These by-products of pretreatment have inhibitory effects on microorganisms [24], and have been found to decrease BNC production [22, 27, 28].

In our previous study, three groups of typical lignocellulose-derived compounds were selected for assessing their influence on *K. xylinus* ATCC 23770 [27, 28]. The three types of compounds studied included aromatic compounds, aliphatic acids, and furan aldehydes. Four of the compounds investigated [furfural, 5-hydroxymethylfurfural (HMF), coniferyl aldehyde, and vanillin] were found to have a negative influence on the growth of the bacterial cells and the yield of BNC, and the bioconversion of these compounds to reduced and oxidized products were reported [27, 28].

Screening of collections of microorganisms gathered from natural or industrial environments can be used to identify strains with high resistance to inhibitors [24]. In this study, several BNC-producing strains were investigated in detail in order to compare their tolerance to typical inhibitors and to evaluate their bioconversion ability. The strains used in this study were *K. xylinus* ATCC 23770 (reference strain) and three other strains, which, according to preliminary experiments, compared favorably with *K. xylinus* ATCC 23770 with respect to BNC production in static cultures. The concentrations of the inhibitors (10 mM furfural, 15 mM HMF, 1.0 mM coniferyl aldehyde, and 2.0 mM vanillin) were chosen on basis of previous studies [27, 28]. The results gave interesting information about inhibitory effects of lignocellulose-derived furan aldehydes and aromatic compounds on the different nanocellulose-producing strains. A better understanding of these effects will benefit selecting the most suitable strains and will facilitate the development of effective processes for production of BNC from lignocellulosics.

## Methods

### Chemicals and microorganisms

**Fig. 1:**
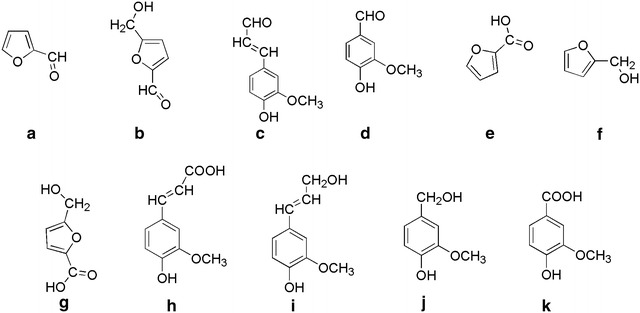
Structures of model inhibitors and related compounds. **a** Furfural, **b** 5-hydroxymethylfurfural, **c** coniferyl aldehyde, **d** vanillin, **e** furoic acid, **f** Furfuryl alcohol, **g** 5-hydroxymethyl-2-furoic acid, **h** ferulic acid, **i** coniferyl alcohol, **j** vanillyl alcohol, and **k** vanillic acid

Reagent-grade chemicals were used in the experiments. Furfural, HMF, coniferyl aldehyde, and vanillin were purchased from Sigma-Aldrich (St Louis, MO, USA). The molecular structural formulae of the compounds and their main conversion products are shown in Fig. 1.

*Komagataeibacter xylinus* ATCC 23770 was obtained from the American Type Culture Collection (Manassas, VA, USA). DHU-ZCY-1 (Z1) was obtained from Hainan Yeguo Foods Co., Ltd, and was deposited as CGMCC 1186 (China General Microbiological Culture Collection Center, Beijing), whereas DHU-ZGD-1 (Z2) and DHU-ATCC-1 (Z3) were mutants of DHU-ZCY-1 and ATCC 23770, respectively. Mutants were obtained through random mutagenesis using chemical and physical standard methods (nitrite impregnation combined with UV radiation).

### Microbial cultures

Bacterial strains were cultivated in 30 mL medium in 100 mL Erlenmeyer flasks. The basal composition of the medium was: 25 g/L glucose, 5 g/L yeast extract, and 3 g/L tryptone. The pH was adjusted to 5.0 with 80% (v/v) sulfuric acid. The concentrations of inhibitors in the medium were: 10 mM furfural, 15 mM HMF, 1.0 mM coniferyl aldehyde, or 2.0 mM vanillin. Aqueous stock solutions of inhibitors with three times as high concentrations as in the cultures were prepared and the pH of the stock solutions was adjusted to 5.0 with either acid (sulfuric acid) or alkali (an aqueous solution of sodium hydroxide). As there was a separate inoculum for each strain and as the growth of the four strains in the medium was slightly different, there were separate control cultures without any inhibitors for each strain.

Care was taken so that the inoculum of each of the four strains (Z1, Z2, Z3, and ATCC 23770) had similar viability. First, a seeding culture for each of the four strains was prepared by transferring a bacterial colony grown on an agar plate into 100 mL of liquid medium without inhibitors. After 36 h of agitated cultivation at 30 °C, the concentration of living cells in the clear culture fluid (no obvious BNC spheres or flocs had yet been formed) was determined. The determination was performed through fluorescence staining using a Live/Dead BacLight Bacterial Viability Kit (Invitrogen, Grand Island, NY, USA) and a Synergy H4 Hybrid Microplate Reader (BioTek Instruments, Winooski, VT, USA), as described in the “Analysis of bacterial viability” section. The glucose concentrations in inocula from seeding cultures were determined by using an Accu-Chek Aviva glucometer (Roche Diagnostics GmbH, Mannheim, Germany). Pre-cultures of each of the strains were made in two steps: (1) A 300 mL mixture with the same concentration of living cells and glucose was prepared by mixing a certain volume of seeding culture, medium, and glucose stock solution. (2) Then, 20 mL of inoculum was introduced into a 100-mL Erlenmeyer flask, which was then incubated at 30 °C with agitation for another 24 h to assure that the bacterial cells were in the exponential growth phase before addition of inhibitory substances was made. After 1 day of cultivation, 10 mL of inhibitor stock solution (or, for controls, 10 mL of autoclaved ultra-pure water) was added to the cultures. Triplicates were performed for each inhibitor and for the control cultures.

The bacterial cultures were incubated statically at 30 °C for 9 days. Samples (2 mL) from each flask were taken aseptically every 2 days during the incubation. The samples were stored at − 20 °C for later analysis.

### Determination of BNC yield

Production of BNC was quantified gravimetrically on basis of dry weight of insoluble BNC obtained at the end of the cultivation. The BNC collected after the incubation was dried to constant weight at 105 °C. The BNC yield on consumed glucose was calculated by using the following equation:

$$BNC\,\, yield\,\, on \,\,consumed \,\,glucose \left( {g/g} \right) = \frac{BNC \left( g \right)}{Glucose \,\,on\,\, the\,\, first\,\, day \left( g \right) - residual\,\, glucose \left( g \right)}$$

The BNC yield as percentage of that of the control (Percentage BNC yield) was calculated by using the following equation:

$$Percentage\,\, BNC \,\,yield \left( \% \right) = \frac{{BNC \,\,volumetric\,\, yield\,\, in \,\,each \,\,culture \left( {\frac{g}{L}} \right)}}{{BNC\,\, volumetric \,\,yield \,\,of \,\,corresponding\,\, control \left( {\frac{g}{L}} \right)}} \times 100$$

### Determination of glucose

The concentration of glucose during the cultivations was monitored by using an Accu-Chek Aviva glucometer (Roche Diagnostics GmbH, Mannheim, Germany). Glucometer buffer containing 8.65 mg/L NaCl, 176.4 mg/L CaCl_2_·2H_2_O, 182.9 mg/L MgCl_2_·6H_2_O, and 201.3 mg/L KCl was prepared, and the pH was adjusted to 7.4 with a solution of NaOH. A standard curve covering the concentration range 0.25–20 mM was made using glucose. Each sample was diluted with the glucometer buffer and the glucose concentration was calculated using the standard curve. The glucose consumption rate (Table 1) was calculated by using the following equation:

$$Glucose\,\, consumption\,\, rate \left( {g/[L \cdot d]} \right) = \frac{{Glucose\,\, on \,\,the\,\, first\,\, day \left( {g/L} \right) - Residual\,\, glucose \left( {g/L} \right)}}{Cultivation \,\,time (d)}$$

**Table 1:** Glucose consumption

Inhibitor	Z1	Z2	Z3	ATCC 23770
(A) Glucose consumption rate on 6th day (g/[L × d])				
10 mM furfural	ND^a^	ND^a^	1.67 ± 0.75	0.50 ± 0.13
15 mM HMF	0.75 ± 0.43	0.78 ± 0.24	1.07 ± 0.29	1.12 ± 0.09
1.0 mM coniferyl aldehyde	2.02 ± 0.25	2.48 ± 0.65	3.59 ± 0.07	2.59 ± 0.07
2.0 mM vanillin	0.73 ± 0.78	0.36 ± 0.53	ND^a^	ND^a^
Control (without inhibitors)	3.02 ± 0.09	3.87 ± 0.23	4.19 ± 0.11	3.07 ± 0.07
(B) Percentage of glucose consumption rate on 6th day (%)				
10 mM furfural	ND^a^	ND^a^	40	16
15 mM HMF	25	20	25	37
1.0 mM coniferyl aldehyde	67	64	86	85
2.0 mM vanillin	24	9	ND^a^	ND^a^
Control (without inhibitors)	100	100	100	100

^a^Not determined

The glucose consumption rate as percentage of that of the corresponding control (Table 1) was calculated by using the following equation:

$$Percentage \,\,glucose \,\,consumption\,\, rate \left( \% \right) = \frac{{Glucose \,\,consumption\,\, rate \,\,in\,\, each\,\, culture \left( {g/[L \cdot d]} \right)}}{{Glucose \,\,consumption \,\,rate \,\,in \,corresponding\,control \left( {g/[L \cdot d]} \right)}} \times 100$$

### Analysis of bacterial viability

The bacterial cells were collected by filtration through Durapore membrane filters (pore size 0.22 μm) in a 1225 sampling manifold (Millipore, Billerica, MA, USA) and were then re-suspended in 8.5 g/L NaCl solution. The viability of the bacterial cells was determined by using a BacLight live/dead bacterial viability fluorescence staining kit (Invitrogen, Grand Island, NY, USA) and a Synergy H4 Hybrid Microplate Reader (BioTek Instruments, Winooski, VT, USA). A standard curve for the relative fluorescence value and the number of bacterial cells was made before the analysis of the samples from the bacterial cultures.

### Analysis of furan derivatives

The concentrations of the furan aldehydes furfural and HMF (Fig. 1) were analyzed using high-performance liquid chromatography (HPLC) with an Agilent series 1200 instrument equipped with a G1315D diode array multiple wavelength detector (Agilent Technologies, Santa Clara, CA, USA) [28]. The column used was a 50 mm × 3.0 mm i.d., 1.8 μm, Zorbax RRHT SB-C18 (Agilent Technologies), and the flow rate was 0.5 mL/min. The column temperature was maintained at 40 °C. Samples (10 μL) were diluted 20-fold with ultrapure water and filtered through 0.2 μm Millex-GN syringe-driven filter units (Millipore). A volume of 2 μL of each diluted sample was injected into the column. Elution was performed with a gradient made of a mixture of ultrapure water and acetonitrile, both of which contained 0.1% formic acid. The gradient started with 3% acetonitrile for 3 min, after which the acetonitrile content increased linearly to 10% after 5 min. For quantitation of the furan aldehydes, standard curves covering the range from 0.5 to 50 ppm were prepared using reference standards. The wavelengths used for quantitation were 280 nm for furfural and 282 nm for HMF.

Potential bioconversion of furfural and HMF to corresponding acids and alcohols was investigated using HPLC-UV–DAD, and retention times and spectra of reference standards for 2-furoic acid (furan-2-carboxylic acid), furfuryl alcohol (2-furanmethanol), and 5-hydroxymethyl-2-furoic acid (Fig. 1) were determined. The wavelength used for quantitation of furoic acid and 5-hydroxymethyl-2-furoic acid was 254 nm, whereas 210 nm was used for furfuryl alcohol.

### Determination of phenolic compounds

The concentrations of the phenols were determined by using the Agilent 1200 series system with the diode array detector and a C18 column (Zorbax SB-C18, 3 × 50 mm, 1.8 μm, Agilent Technologies). The column temperature was maintained at 40 °C. Samples were diluted with ultrapure water and filtered using 0.2 μm syringe-driven filter units (Millex-GN). Ten microlitres of diluted sample were injected into the column and were eluted for 40 min at a flow rate of 0.4 mL/min. The eluent consisted of a gradient of ultrapure water and acetonitrile, both of which contained 2 mM formic acid. The concentration of acetonitrile was increased from 0 to 5% within the first 5 min, and further on to 10% after 10 min. After 20 min, the concentration of acetonitrile was 30%, and it was then raised to 50% after 30 min. At the end of the 40 min period, the concentration of acetonitrile was decreased to 5%.

Standard curves for the phenols used in the experiments were prepared within the concentration range 0.5–100 ppm. The wavelengths used for quantitation were: 254 or 330 nm for coniferyl aldehyde, 280 nm for vanillin, vanillyl alcohol, and vanillic acid, 280 or 330 nm for ferulic acid, and 254 nm for coniferyl alcohol.

## Results and discussion

### Impact of inhibitors on bacterial strains

**Fig. 2:**
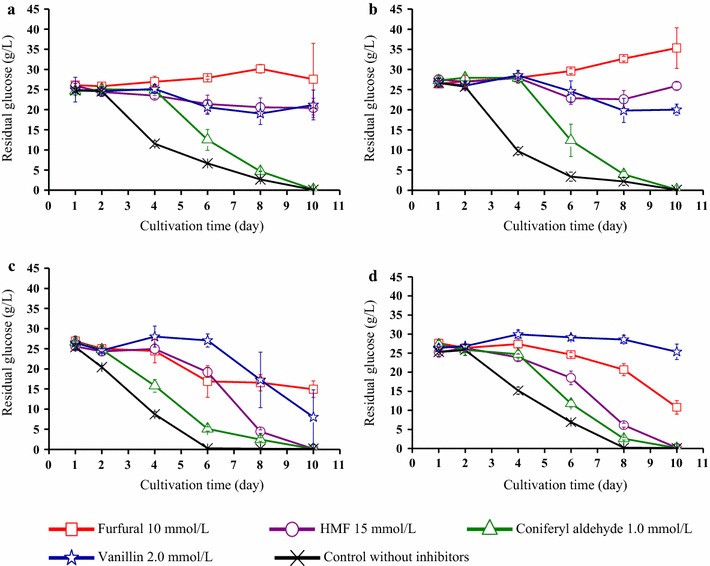
Residual glucose concentrations in cultures containing inhibitors: **a** Z1 strain, **b** Z2 strain, **c** Z3 strain, and **d** ATCC 23770 strain

Figure 2 and Table 1 show the effects of the inhibitors on the glucose consumption of the four strains. As the growth characteristics of the strains differ, and perhaps also the conditions of the pre-cultures, it should be useful to compare the effects of the inhibitors by investigating the percentage of glucose consumption in relation to the control of the same strain, as in Table 1. Glucose consumption was calculated on the 6th day (Table 1), before the sugar in any of the cultures was exhausted. On the 6th day, the glucose in control cultures of Z3 was almost consumed (Fig. 2c), which resulted in the highest glucose consumption rate among the four strains (Table 1a). As expected on basis of previous studies, the concentrations of the four inhibitors were sufficient to inhibit ATCC 23770 (Fig. 2d).

Z1 and Z2 were strongly affected by 10 mM furfural and no glucose consumption could be detected. The slight increase in glucose concentration during cultivation of Z1 and Z2 in the presence of furfural (Fig. 2a and b) can be attributed to absence of glucose consumption combined with some evaporation of water from the culture medium. In the presence of 10 mM furfural, Z3 had consumed more glucose than the other three strains by day 6 (Table 1), but the glucose consumption slowed down at the end of the cultivation (Fig. 2c). In contrast, cultures of ATCC 23770 exhibited rapid glucose consumption at the end of the cultivation in furfural-containing medium (Fig. 2d). By the 6th day, 15 mM HMF had an obvious inhibitory effect on all four strains (Table 1). Cultures of ATCC 23770 exhibited the highest glucose consumption (37% of that of the control) in the presence of HMF, while glucose consumption for the other three strains was ≤ 25% of that of the corresponding control (Table 1b). At the end of the cultivations, Z3 and ATCC 23770 performed well in the presence of HMF, whereas Z1 and Z2 did not (Fig. 1). Taken together, the results indicate that Z3 and ATCC 23770 are more tolerant to furan aldehydes than the other strains. Considering the structural similarities of furfural and HMF (Fig. 1) it is not surprising that the effects on the strains show similarities.

With regard to coniferyl aldehyde, the effects on ATCC 23770, Z1 and Z2 were similar as there was an initial lag phase that lasted for about 4 days (Fig. 2). Z3 was the only strain that exhibited strong sugar consumption already after 4 days (Fig. 2c). Z3 and ATCC 23770 exhibited the highest glucose consumption rate (Table 1b). The resistance to vanillin was difficult to compare, as the strains responded in different ways. Z1 and Z2 exhibited some consumption of glucose during the first 6 days of the cultivations (Fig. 2) and therefore they had the highest glucose consumption rates (Table 1). After that, Z1 and Z2, did not perform well (Fig. 2). ATCC 23770 and particularly Z3 performed better at the end than at the beginning (Fig. 2). After 12 days growth in vanillin-supplemented medium, Z3 exhibited by far the best glucose consumption among the four strains (Fig. 2). Thus, the sugar consumption analysis consistently points towards Z3 performing best in the presence of phenolic inhibitors.

**Fig. 3:**
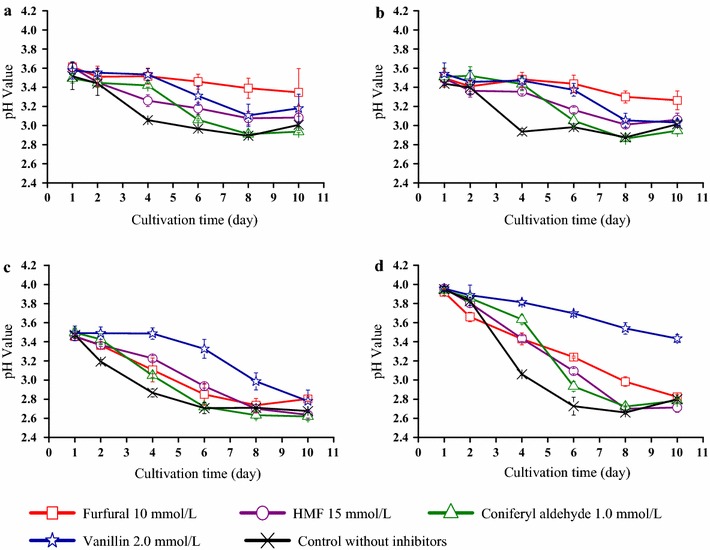
Changes in pH of the cultures of **a** Z1 strain, **b** Z2 strain, **c** Z3 strain, and **d** ATCC 23770 strain

Figure 3 shows the pH of the medium. At day 1, the pH had already dropped from the initial value 5.0 to 3.5–4.0. At the end of the cultivations, the pH had in most cases dropped to around 3.0 or even lower than 3.0 (Fig. 3). Cultures with low glucose consumption, for example Z1 and Z2 with furfural and ATCC 23770 with vanillin (Fig. 2, Table 1), also exhibited a lower drop in pH (Fig. 3). This was evidently a consequence of lower metabolic activity, as BNC-producing bacteria can consume glucose and produce acids [29]. In addition, bioconversion of furan aldehydes and phenolic aldehydes by oxidation could potentially result in the formation of the corresponding carboxylic acids, which then could contribute to acidification. Bioconversion of inhibitors is further addressed in a subsequent section.

**Fig. 4:**
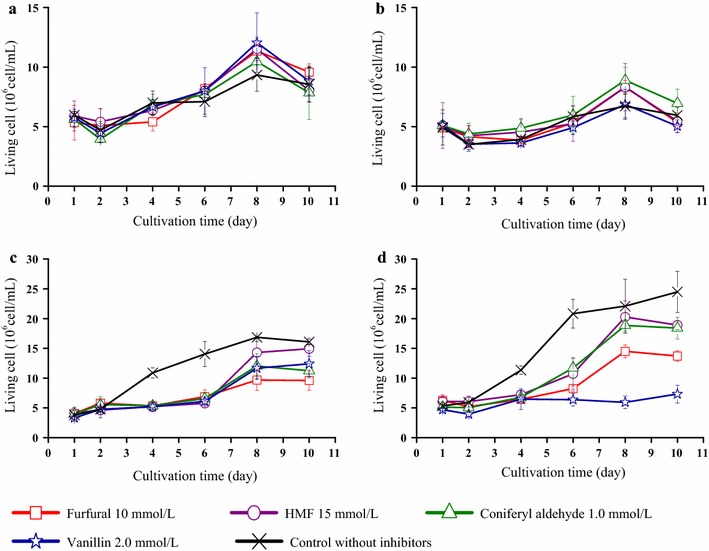
Concentrations of living cells in cultures of **a** Z1 strain, **b** Z2 strain, **c** Z3 strain, and **d** ATCC 23770 strain

The concentrations of living cells during the cultivations are shown in Fig. 4. In a static cultivation of BNC-producing bacteria, the cells would be divided between the cellulose phase and the liquid phase. The data in Fig. 4 represent the concentrations of living cells in the liquid phase, as determination of living cells embedded in the cellulose network would not be very efficient and as a complicated handling process would not be suitable for real-time monitoring of the cells. The initial concentrations were around 5 × 10^6^ cells/mL (Fig. 4). The highest values of the control cultures were 24.5 × 10^6^ cells/mL for ATCC 23770, 16.9 × 10^6^ cells/mL for Z3, 9.3 × 10^6^ cells/mL for Z1, and 6.7 × 10^6^ cells/mL for Z2.

It is possible to dissolve BNC using cellulases for counting the number of living cells, but in the current study there would have been practical problems with that approach. Temperatures of around 50 °C are needed for efficient hydrolysis of cellulose, real-time determination of cell numbers is a great advantage, and partially degraded BNC results in background fluorescence. A pre-experiment suggested that none of the bacterial strains used in this work were able to survive during cultivation at higher temperatures than around 30 °C, a temperature that is low with respect to efficient hydrolysis of cellulose using commercial cellulase preparations. Furthermore, the main advantage of using the fluorescence staining method was to make it possible to determine the numbers of living cells in real time. Complete dissolution of BNC by cellulases would be too time-consuming to be suitable for instant counting of the numbers of living cells. A preliminary attempt to use cellulases to dissolve the BNC membrane indicated that it was difficult to dissolve the membrane completely within a reasonable time period. Furthermore, the partially dissolved cellulose gave a strong background fluorescence since the cellulose fragments nonspecifically adsorbed the staining dyes.

Control cultures of Z3 and ATCC 23770 typically had higher concentrations of free-living cells than corresponding cultures with inhibitors (Fig. 4). For Z1 and Z2, which exhibited poorer growth, it was difficult to discern any difference between control cultures and cultures with inhibitors (Fig. 4). Further research is needed to quantify the fraction of living cells embedded in the cellulose layer, and the distribution of living cells in the liquid phase and in the cellulose.

Table 2 shows the yield of BNC on consumed glucose, the volumetric yield of BNC, and the volumetric yield of BNC for cultures with inhibitors in relation to the yield of the corresponding control cultures. The Z3 control cultures gave a volumetric BNC yield of 14.8 g/L, which was the highest among the four strains, and it also had the highest BNC yield on consumed glucose (0.59 g/g). With a volumetric BNC yield of 8.7 g/L and a yield on consumed glucose of 0.34 g/g, ATCC 23770 was the least productive strain. The BNC yields of Z1 and Z2 were similar, with a volumetric yield of about 13 g/L and a yield on consumed sugar of about 0.5 g/g. These results clearly show the potential of strains Z1, Z2, and Z3 in relation to the well-studied ATCC 23770, and especially the superior performance of Z3.

**Table 2:** Yields of bacterial nanocellulose

Strain	Z1	Z2	Z3	ATCC 23770
A. Bacterial nanocellulose yield on consumed glucose (g/g)				
10 mmol/L furfural	^#1^	^#1^	0.76 ± 0.10	0.29 ± 0.02
15 mmol/L HMF	^#1^	^#1^	0.47 ± 0.02	0.34 ± 0.03
1.0 mmol/L coniferyl aldehyde	0.55 ± 0.01	0.52 ± 0.01	0.56 ± 0.02	0.38 ± 0.03
2.0 mmol/L vanillin	^#1^	^#1^	0.80 ± 0.10	^#1^
Control (without inhibitors)	0.52 ± 0.01	0.49 ± 0.01	0.59 ± 0.02	0.34 ± 0.03
B. Volumetric yield of bacterial nanocellulose (g/L)				
10 mM furfural	3.03 ± 1.45	3.93 ± 0.95	8.96 ± 0.96	4.85 ± 0.23
15 mM HMF	9.81 ± 1.36	10.62 ± 0.24	11.95 ± 0.44	8.59 ± 0.55
1.0 mM coniferyl aldehyde	13.37 ± 0.16	14.30 ± 0.27	14.81 ± 0.53	10.33 ± 0.77
2.0 mM vanillin	10.18 ± 1.99	11.73 ± 0.42	13.95 ± 0.74	5.76 ± 0.73
Control (without inhibitors)	12.82 ± 0.18	12.93 ± 0.49	14.78 ± 0.43	8.65 ± 0.83
C. Percentage BNC yield (%)				
10 mM furfural	24	31	60	56
15 mM HMF	77	82	81	99
1.0 mM coniferyl aldehyde	104	111	101	119
2.0 mM vanillin	79	91	94	67
Control (without inhibitors)	100	100	100	100

^#1^Glucose consumption close to zero

With 10 mM furfural in the medium, the volumetric BNC yield of Z1 and Z2 dropped to 3–4 g/L, which was less than one third of the corresponding control cultures (Table 2). Z3 and ATCC 23770 where much less affected by the furfural, and exhibited volumetric BNC yields that were 56–60% of those of the corresponding controls (Table 2). These results agree well with the sugar consumption data in Fig. 2 and Table 1. Section B in Table 2 shows that Z1 and Z2 with furfural were the only cases where any of the strains Z1, Z2, and Z3 performed worse than the corresponding cultures with ATCC 23770, again highlighting the very good BNC-producing capability of the three new strains.

Inclusion of 15 mM HMF in the medium decreased the volumetric productivity of Z1, Z2, and Z3 to about 80% of that of the corresponding control cultures (Table 2). However, the volumetric productivities for all three strains were still higher than that of ATCC 23770 cultures with HMF.

Addition of 1 mM coniferyl aldehyde to the medium did not have any negative effect on the BNC production for any of the strains (Table 2). For some strains, the BNC production was even slightly enhanced in the presence of coniferyl aldehyde (Table 2), but this increase was not significant (*P* > 0.05) compared to the control.

Addition of 2 mM vanillin resulted in volumetric BNC yields that were 67–94% of those of the corresponding control cultures (Table 2). It is noteworthy that 2 mM vanillin had quite severe effect on the sugar consumption of strains Z1, Z2, and ATCC 23770, as more than two thirds of the sugar remained (Fig. 2, Table 1), and that only Z3 consumed most of the sugar. Despite the poor sugar consumption of Z1, Z2, and ATCC 23770, they still produced at least two thirds of the BNC of the inhibitor-free controls.

In summary, Z3 gave the highest yield of BNC and was most tolerant against the inhibitors. ATCC 23770 was the least productive strain though it exhibited better resistance to furan aldehydes, especially furfural, than Z1 and Z2. The strains Z1 and Z2 had more tolerance than ATCC 23770 to vanillin, but were more sensitive to furfural.

### Bioconversion of inhibitors

Samples taken at the beginning and at the end of the cultivations were analyzed and the yields of bioconversion products of inhibitors are shown in Table 3. The main bioconversion product of furfural was furoic acid. Z3 and ATCC 23770 displayed a higher fraction of furoic acid at the end of the cultivations (71–78%) than Z1 and Z2 (38–51%). 5-Hydroxymethyl-2-furoic acid was detected as the predominant bioconversion product of HMF. Also for 5-hydroxymethyl-2-furoic acid, Z3 and ATCC23770 displayed a higher fraction of the main bioconversion product (95–100%) than Z1 and Z2 (49%). Previous work [28] indicated that furfural and HMF were oxidized by *K. xylinus* ATCC 23770, and that the predominant bioconversion products of these two aldehydes were furoic acid and 5-hydroxymethyl-2-furoic acid, respectively. The current study shows that this product pattern is valid also for three other BNC-producing strains. With addition of 10 mM furfural, furoic acid was formed in cultures of Z1 and Z2 even though there was no clear sign of glucose consumption. A similar phenomenon was observed in previous work [28] with the strain *K. xylinus* ATCC 23770. Glucose consumption and cell growth of ATCC 23770 were almost totally inhibited when the concentration of furfural and HMF was higher, but bioconversion of furan aldehyde could still occur [28]. In our previous study, it was hypothesized that *K. xylinus* ATCC 23770 oxidizes furfural and HMF in reactions involving the reduction of NAD(P)^+^ [28]. It is noteworthy in this context that a related bacteria, *Acetobacter aceti*, has been found to be capable of conversion of polyconjugated aldehydes into their corresponding acids due to membrane-bound dehydrogenases [30]. The acetic acid bacterium *Gluconobacter oxydans* has the ability to catalyze regio- and stereo-selective oxidation of a great variety of alcohols, polyols, carbohydrates, and related compounds [31]. It is being used for the production of l-sorbose, d-gluconic acid, dihydroxyacetone, and precursors of vitamin C and in many other biotechnological processes [32]. This oxidation is catalyzed by various membrane-bound dehydrogenases [33]. The membrane-bound aldehyde dehydrogenase of *G. oxydans* has been reported to mainly oxidize acetaldehyde to acetate [34, 35]. Oxidation of furan aldehydes by BNC-producing strains, as observed in this work, might also be related to membrane-bound dehydrogenases.

**Table 3:** Yields of bioconversion products from inhibitors

Inhibitors	Furfural	HMF	Coniferyl aldehyde	Vanillin	
Main bioconversion products	Furoic acid (%)	5-Hydroxymethyl-2-furancarboxylic acid (%)	Ferulic acid (%)	Vanillyl alcohol (%)	Vanillic acid (%)
Z1	38	49	36	13	10
Z2	51	49	31	17	12
Z3	71	100	35	80	14
ATCC 23770	78	95	38	28	12

The table shows the fractions of conversion products based on the initial amounts of inhibitors. Conversions below 2.5% are not shown

As shown in Table 3, the main bioconversion product of coniferyl aldehyde was the oxidation product ferulic acid. The fraction of ferulic acid was similar for the four strains ranging from 31 to 38%. With regard to vanillin, the reduction product vanillyl alcohol was predominant (13–80%), although the oxidation product vanillic acid was also common (10–14%) (Table 3). Z3 produced by far the largest amounts of vanillyl alcohol and the ratio vanillyl alcohol: vanillic acid was 5.7, much higher than the other strains that exhibited ratios ranging from 1.3 to 2.3. In previous work with *K. xylinus* ATCC 23770 and coniferyl aldehyde and vanillin, small amounts of coniferyl alcohol and very low concentration of vanillic acid were also detected in the cultures while ferulic acid and vanillyl alcohol were the predominant products [27]. It might be considered surprising that for one of the phenolic aldehydes, coniferyl aldehyde, the oxidation product ferulic acid was predominant, whereas for the other, vanillin, the reduction product vanillyl alcohol was predominant, but through the current study this finding is now supported by analysis of three other BNC-producing bacteria.

In a study of the bacterium *Pseudomonas* sp. strain HR 199, the oxidation of coniferyl aldehyde to ferulic acid was found to be catalyzed by NAD^+^-dependent coniferyl aldehyde dehydrogenase (CALDH), which is encoded by the gene *calB* [36]. Kubiak et al. [37]. investigated the complete genome sequence of *G. xylinus* E25 and identified a mega plasmid which might be important for the survival of the strain in unfavorable environments. Some *G. xylinus* E25 genes connected to oxidoreductases (for instance H845_1089 and H845_1144) and some genes connected with cell defense mechanisms (five efflux systems components and eight other transporters) were identified. Proteins coded for by such genes may help to explain the ability of *G. xylinus* to grow in media containing aromatic compounds [37].

## Conclusions

The resistance of four BNC-producing strains to selected lignocellulose-derived inhibitors consisting of two furan aldehydes and two aromatic aldehydes was investigated. Z3 and ATCC 23770 exhibited better resistance to furan aldehydes, especially furfural, than Z1 and Z2. Z3 exhibited better resistance to aromatic aldehydes than the other strains. The data also show that Z3, Z2, and Z1 have potential to give superior BNC yields compared to the commonly used strain ATCC 23770. On the basis of the BNC yields obtained and to a better understanding of the inhibitor tolerance of the strains, Z3 emerges as the most suitable candidate strain for BNC production using lignocellulosic hydrolysates. The four BNC-producing strains exhibited strong similarities with regard to predominant bioconversion products from the inhibitors, but displayed different capacity to convert the inhibitors, which may be related to the differences in inhibitor tolerance. Identification of bacterial genes encoding oxidoreductases that partake in the bioconversion and detoxification of inhibitors is an interesting area for further studies.
